# Comparative Study of Exome Copy Number Variation Estimation Tools Using Array Comparative Genomic Hybridization as Control

**DOI:** 10.1155/2013/915636

**Published:** 2013-11-04

**Authors:** Yan Guo, Quanghu Sheng, David C. Samuels, Brian Lehmann, Joshua A. Bauer, Jennifer Pietenpol, Yu Shyr

**Affiliations:** ^1^Center for Quantitative Sciences, Vanderbilt University, Nashville, TN 37027, USA; ^2^Center for Human Genetics Research, Vanderbilt University, Nashville, TN 37037, USA; ^3^Department of Biochemistry, Vanderbilt University, Nashville, TN 37027, USA

## Abstract

Exome sequencing using next-generation sequencing technologies is a cost-efficient approach to selectively sequencing coding regions of the human genome for detection of disease variants. One of the lesser known yet important applications of exome sequencing data is to identify copy number variation (CNV). There have been many exome CNV tools developed over the last few years, but the performance and accuracy of these programs have not been thoroughly evaluated. In this study, we systematically compared four popular exome CNV tools (CoNIFER, cn.MOPS, exomeCopy, and ExomeDepth) and evaluated their effectiveness against array comparative genome hybridization (array CGH) platforms. We found that exome CNV tools are capable of identifying CNVs, but they can have problems such as high false positives, low sensitivity, and duplication bias when compared to array CGH platforms. While exome CNV tools do serve their purpose for data mining, careful evaluation and additional validation is highly recommended. Based on all these results, we recommend CoNIFER and cn.MOPs for nonpaired exome CNV detection over the other two tools due to a low false-positive rate, although none of the four exome CNV tools performed at an outstanding level when compared to array CGH.

## 1. Introduction

Next-generation sequencing technology, piloted by the Illumina platform, has substantially decreased the cost of sequencing on large genomic regions. However it is still financially prohibitive to perform whole genome sequencing on a large number of subjects, especially for large scale genetic epidemiology association studies, at a sufficient depth for accurate genotype calls. The human exome represents about 1–3% of the human genome with approximately 30–50 million base pairs but accounts for over 85% of all mutations identified in Mendelian disorders [1]. As a result, exome sequencing is currently an attractive and practical approach for investigating coding variations.

Exome sequencing is typically used to identify single nucleotide polymorphisms (SNPs), somatic mutations (through paired sample comparison), and small and large structural variations. A lesser-known application of exome sequencing data is to identify copy number variations (CNV). CNVs are a structural variation in which cells have an abnormal number of copies of one or more sections of the DNA. Normal cells are diploid containing two copies of DNA and abnormal CNVs refer to large regions of the chromosome that have been deleted or duplicated. CNV characterization is important for both the basic understanding of many diseases and their diagnoses. CNVs have been linked to various diseases including autism [2], obesity [3], breast cancer [4], colorectal cancer [5], and lung cancer [6].

Traditionally, CNV detection has been performed with cytogenetic techniques such as fluorescent in situ hybridization, array comparative genomic hybridization (array CGH), and with virtual karyotyping using SNP arrays. Array CGH is commonly considered to be a reliable method for discovering novel CNVs because of the relatively even distribution of probes [7]. Many high-impact copy number studies [8–10] were based on results derived from array CGH methods. 

Whole genome sequencing data are relatively even in coverage, thus making it ideal for CNV discovery. Many CNV methods [11–17] have been developed for whole genome sequencing data. On the other hand, exome sequencing's depth is strongly affected by the enrichment regions, thus making it less ideal for CNV discovery. However, given the popularity of exome sequencing and the massive amount of exome sequencing data accumulated thus far, there is much interest in inferring CNVs from exome sequencing data. Thus, multiple CNV tools targeting exome sequencing data have been developed. We have cataloged sequencing data based CNV tools in Table S1 in the Supplementary Material available online at http://dx.doi.org/10.1155/2013/915636.

To determine if exome sequencing could provide reliable CNV detection, we performed array CGH and exome sequencing on 16 breast cancer cell lines. The data obtained from this study provides us an opportunity to evaluate the CNV discovery method based on exome sequencing while using array CGH as the reference. To date, there have been seven CNV tools targeting exome sequencing data: ExomeCNV [18], CoNIFER [19], cn.MOPS [20], exomeCopy [21], ExomeDepth [22], CNANorm [23], and CONTRA [24]. CNANorm, CONTRA, and ExomeCNV are specifically designed for paired tumor and normal samples. The other four do not require paired sample as input. These four tools cover a unique aspect of exome sequencing data. As exome sequencing become more commercially affordable, large epidemiology studies which only have blood samples available are more likely to choose exome sequencing over SNP array. The unpaired exome CNV tools will become the only suitable tools for CNV analysis. In this study, we systematically evaluated the performance of these four tools against each other using array CGH as the reference. We present our findings in detail and make a recommendation for the best unpaired exome CNV discovery tool based on our findings.

## 2. Materials and Methods

We performed array CGH on 16 breast cancer cell lines (Table S2) using the Agilent SurePrint G3 Human CGH Microarray Kit. This array CGH kit contains 963,029 distinct probes with 2.1 KB overall median probe spacing. The array CGH chips were scanned using the GenePix 4000B scanner, and probe intensities were normalized using Agilent's Feature Extraction software. CNVs were called using the Aberration Detection Method 2 (ADM2), a very broadly used CNV detection method for array CGH platform through GeneSpring Software. Exome sequencing data analysis was also performed on the same 16 breast cancer cell lines using Illumina's TrueSeq exome enrichment kit on Illumina's HiSeq 2000 platform. The sequencing reads are pair end 75 base pair long. The pooled, barcoded raw data produced by the Illumina HiSeq 2000 high-throughput sequencer was first split using barcode splitting software to obtain raw data for each individual. The raw data were aligned using BWA [25], which was designed based on the Borrows-Wheeler Transformation. The Human reference genome HG19 was used for alignment. The aligned BAM [26] files were locally realigned using the Genome Analysis Toolkit (GATK) [27] developed by the Broad Institute. The local realignment step aims to correct misalignment caused by the presence of insertions or deletions (indels). To further increase the local realignment accuracy, after local realignment, we performed base quality score recalibration on the realigned BAM files using GATK's recalibration tool. The recalibration tool attempts to correct for variations in quality with machine cycle and sequence context. The resulting BAM files contain not only more accurate base quality scores but also more widely dispersed ones. The recalibrated BAM files were filtered by removing all reads with mapping quality Phred score [28] less than 20 and all bases with base quality Phred score less than 20 (meaning that the probability of the base call being wrong is less than 0.01). CNVs on the processed BAM files were called using CoNIFER, cn.MOPS, exomeCopy, and ExomeDepth. Each of the four tools provides a wide range of parameters. We either consulted with the authors of the tools for the best parameters or used the author recommended parameters for the analysis. The exact command line used for each tool is listed in Table S3. Results of CNV detection from these four tools were compared to the array CGH results to determine the strength and weakness of each program.

## 3. Results and Discussion

### 3.1. Results

We generated high quality exome sequencing data using the Illumina TrueSeq enrichment kit on the HiSeq 2000 platform. All samples' raw data passed the initial quality control using FASTQC. On average, each sample had 117 million (range: 73 to 183 million) reads sequenced. The average capture efficiency was 48% (range: 39% to 62%). No notable quality issues were observed for the exome sequencing data (Table S2).

**Figure 1:**
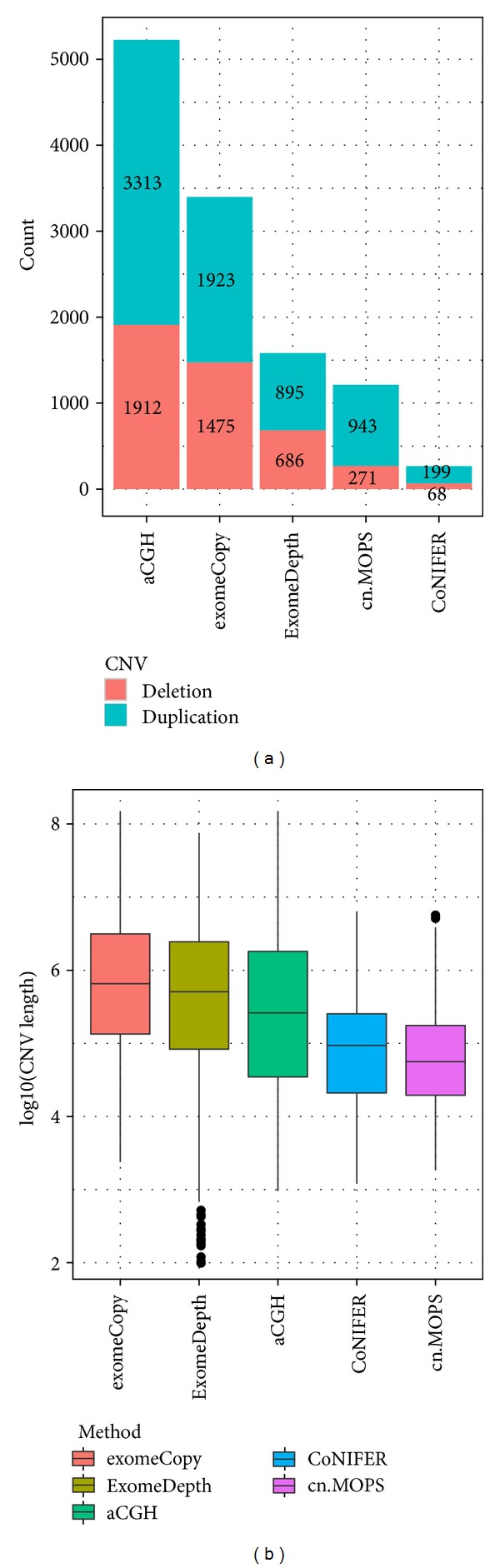
Overview of the CNVs detected by array CGH and four algorithms. (a) Barplot of the duplication and deletion CNVs detected by five methods. (b) Boxplot of the CNV length detected by five methods.

Across all 16 samples, array CGH identified 5,225 CNVs. Among the four exome CNV tools, exomeCopy identified the most CNVs (3,398), and CoNIFER identified the least (267). ExomeDepth (1,581) and cn.MOPS (1,214) identified a moderate number of CNVs (Figure 1(a)). The median CNV length identified by array CGH was 261,400 base pairs (range: 959 to 146,900,000 base pairs). ExomeDepth and exomeCopy identified the CNVs with longer average length than did array CGH, while CNVs identified by CoNIFER and cn.MOPS had shorter average length compared to array CGH (Figure 1(b)). 

**Figure 2:**
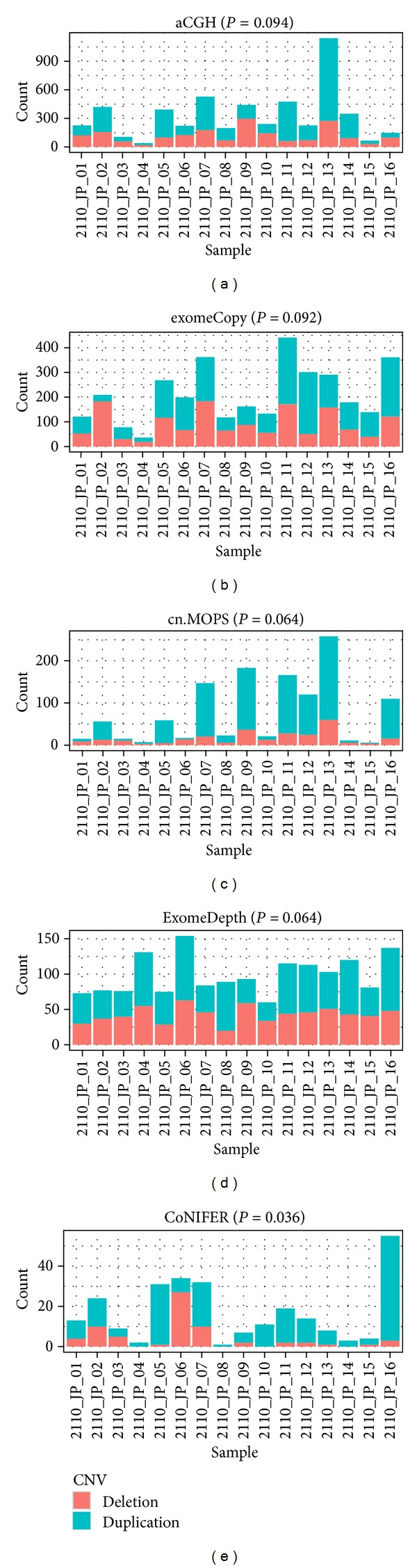
Barplot of duplication and deletion CNVs detected from each sample by five methods. The *P* value beside each method name was calculated by paired Wilcoxon signed rank tests following FDR correction. It indicated the detection bias between duplication and deletion CNVs of that method. Array CGH and exomeCopy showed unbiased duplication and deletion while CoNIFER had strong bias toward duplication. Cn.MOPS and ExomeDepth showed marginal bias toward duplication.

We also determined the deletion-duplication ratio for array CGH and the four exome CNV tools by sample. Each sample has distinct molecular characteristics that result in distinct deletion and duplication ratios. Consistently observing more duplication than deletions or vice versa across all samples may be an indication of an algorithm-specific bias. For array CGH, across all 16 samples, we observed 9 samples with more duplication and 7 samples with more deletions, a rather ideal scenario (Figure 2(a)). For exomeCopy and cn.MOPS, we observed 10 samples with more duplication and 6 samples with more deletions (Figures 2(b) and 2(c)). For ExomeDepth, we observed 11 samples with more duplication and 5 samples with more deletions (Figure 2(d)). For CoNIFER, we observed 14 samples with more duplication and 2 samples with more deletions (Figure 2(e)). We conducted paired Wilcoxon signed rank tests to see if there is any duplication or deletion bias. We found that array CGH showed unbiased duplication and deletions with *P* value = 0.11, exomeCopy also showed unbiased duplication and deletions with *P* value = 0.1. CoNIFER had strong bias toward duplication with *P* values equal to 0.025. ExomeDepth and Cn.MOPS showed marginal bias toward duplication with *P* value = 0.064. To identify the exome CNV tool with the most similar deletion duplication ratio, we conducted pairwise Kullback-Leibler divergence distance on both duplication and deletions proportions (Table 1). The values in Table 1 are measures of the difference between the tested method and the array CGH method, with smaller values indicating less difference. For both duplication and deletions, cn.MOPS showed the shortest distance to array CGH, with 0.15 for deletions and 0.36 for duplication. 

**Figure 3:**
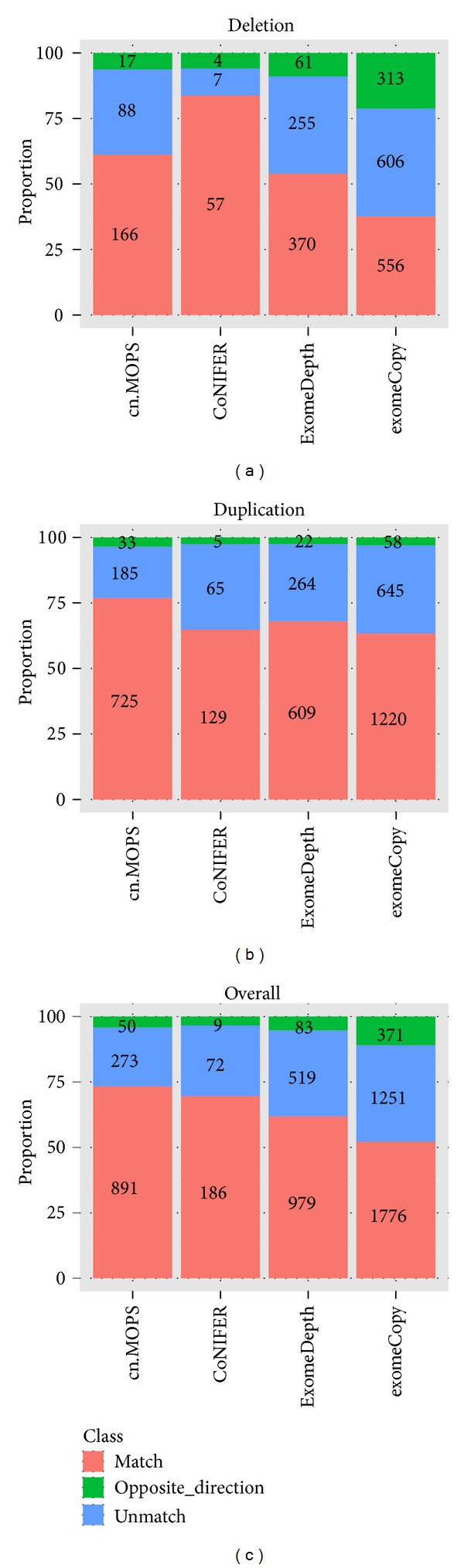
Specificity of four algorithms for CNV detection. CoNIFER identified many fewer CNVs but with a high true positive rate at deletion detection. ExomeDepth and ExomeCopy showed comparable specificity with CoNIFER on duplication detection but many more false positives on deletion detection. Cn.MOPS showed best specificity at duplication detection and second best specificity at deletion identified many more CNVs than CoNIFER. Overall, cn.MOPS achieved the highest specificity among all four algorithms.

To measure the consistency with array CGH, we determined the overlap of CNVs identified between each of the exome CNV tools with array CGH. Overlapping CNVs were defined as regions that share at least 50% of their base pairs. We also used a less strict option where two CNVs are considered consistent if only 1% of the base pairs overlapped. However, regardless of which option we use, the results were very similar, since if two CNVs from two methods overlapped, most of them overlapped by at least 50% (Table S4). Compared to the array CGH platform, cn.MOPS had the best true positive rate for duplication with 76.9%, and CoNIFER had the best true positive rate for deletions with 83.8% (Figure 3). ExomeDepth and exomeCopy had comparable true positive rates for duplication with CoNIFER but lower true positive rates for deletions. Also interestingly, all four exome CNV tools identified some CNVs with opposite direction (deletion instead of duplication or vice versa) compared to array CGH. ExomeDepth and exomeCopy had relatively low proportion of CNVs with opposite direction on duplication (2.5% and 3.0%) but a moderate proportion of CNVs with opposite direction on deletion (8.9% and 10.9%), while cn.MOPS and ConNIFER had a relatively low proportion of CNVs with opposite direction (3.5% and 2.5% for duplication, 6.3% and 5.9% for deletion). CoNIFER and cn.MOPS detected CNVs with a much lower false-positive rate. In such a scenario, CoNIFER and cn.MOPS are much more desirable, because it is impossible to tell true-positives from false-positives without any prior knowledge. ExomeDepth and exomeCopy also demonstrated comparable performance for detecting duplication with CoNIFER.

### 3.2. Discussion

Exome sequencing is widely used to conduct genomic research. Identifying CNVs through exome sequencing data has been a popular topic over the last few years. Compared to array-based methods, identifying CNVs through exome sequencing data has some shortcomings. First, the exons within the genome are not evenly placed. They are located at fixed positions, unlike probes which can be designed to be placed evenly across the whole genome. Thus if only depth information from unevenly located exons can be used for CNV assessment then CNV detection over a long intergenic region would be unreliable. Also, a probe can be designed to avoid hybridization at problematic genomic regions such as regions with high GC content. A high GC content region can affect the sequencing depth for exome sequencing, which makes identifying CNVs using exome sequencing data even more complicated. Additional normalization to correct noise caused by effects such as GC content is desirable. 

 With these known difficulties, many exome CNV tools have been developed over the last few years. In this study, we evaluated the effectiveness of four popular unpaired exome CNV tools: cn.MOPS, CoNIFER, ExomeDepth, and exomeCopy using 16 breast cancer cell lines. We identified CNVs using these four tools and verified the results against array CGH results from the same samples. CoNIFER and cn.MOPS identified much fewer CNVs but with a high true-positive rate. ExomeDepth and exomeCopy produced comparable performance for duplication detection with CoNIFER. In terms of duplication-deletion proportion, we found that with the exception of exomeCopy, the exome CNV tools showed a significant bias toward duplication. This could be the result of an artifact of the exome CNV algorithm that underestimates the normal copy number based on depth. Using the Kullback-Leibler divergence distance, we found that cn.MOPS is the closest to array CGH in terms of duplication or deletion proportion across samples. Based on all these results, we recommend CoNIFER and cn.MOPS for nonpaired exome CNV detection over the other two tools due to a low false-positive rate, although none of the four exome CNV tools performed at an outstanding level when compared to array CGH. In summary, there is value in identifying CNVs using exome sequencing data but extra caution needs to be taken into consideration due to the high false positive rate. Identifying CNVs is almost never the primary goal of the exome sequencing study, and it should stay that way due to the noise introduced by exome sequencing data. Identifying CNVs using exome sequencing data is potentially a good secondary data mining technique. Based on our comparison of the methods, results generated from exome CNV tools should be evaluated thoroughly, and additional validation is highly recommended to eliminate false-positives and to ensure quality data.

## 4. Conclusions

Using array CGH result as control, we systematically compared four popular exome CNV tools (CoNIFER, cn.MOPS, exomeCopy, and ExomeDepth) on exome sequencing data generated from 16 breast cancer cell lines. Among evaluated four tools, we recommend CoNIFER and cn.MOPS for nonpaired exome CNV detection due to a low false-positive rate. Our results suggest that exome CNV tools are subjected to high false positive rat, low sensitivity, and duplication bias when compared to array CGH platform. Thus careful evaluation and additional validation is highly recommended.

## Supplementary Material

Table S1: CNV Tools Summary

## Figures and Tables

**Table 1:** Kullback-Leibler test on similarity with array CGH.

	aCGH	cn.MOPS	exomeCopy	ExomeDepth	CoNIFER
Deletion CNVs proportion similarity					
aCGH	0	0.14	0.16	0.17	2.24
cn.MOPS	0.15	0	0.22	0.24	1.84
exomeCopy	0.16	0.23	0	0.2	1.88
ExomeDepth	0.22	0.27	0.23	0	2.56
CoNIFER	0.8	0.97	0.87	0.72	0

Duplication CNVs proportion similarity					
aCGH	0	0.4	0.4	0.47	0.66
cn.MOPS	0.36	0	0.42	0.66	0.61
exomeCopy	0.42	0.59	0	0.18	0.26
ExomeDepth	0.55	1.06	0.25	0	0.55
CoNIFER	0.77	0.58	0.26	0.42	0
