# Corrigendum to “Comparative Study of Exome Copy Number Variation Estimation Tools Using Array Comparative Genomic Hybridization as Control”

**DOI:** 10.1155/2017/7409598

**Published:** 2017-07-18

**Authors:** Yan Guo, Quanhu Sheng, David C. Samuels, Brian Lehmann, Joshua A. Bauer, Jennifer Pietenpol, Yu Shyr

**Affiliations:** ^1^Center for Quantitative Sciences, Vanderbilt University, Nashville, TN 37027, USA; ^2^Center for Human Genetics Research, Vanderbilt University, Nashville, TN 37037, USA; ^3^Department of Biochemistry, Vanderbilt University, Nashville, TN 37027, USA

In the article titled “Comparative Study of Exome Copy Number Variation Estimation Tools Using Array Comparative Genomic Hybridization as Control” [1], the name of the second author was given incorrectly as Quanghu Sheng. The author's name should have been written as Quanhu Sheng. The revised authors' list is shown above.
